# Increased serum triglycerides and reduced HDL cholesterol in male rats after intake of ammonium chloride for 3 weeks

**DOI:** 10.1186/1476-511X-12-92

**Published:** 2013-06-25

**Authors:** Arne Torbjørn Høstmark, Marianne Sylvana Haug Lunde, Anna Haug

**Affiliations:** 1 Institute of Health and Society, University of Oslo, Norway, Box 1130 Blindern, 0318, Oslo, Norway; 2 Current affiliation is Eli Lilly AS, Grenseveien 99, P.O. Box 6090 Etterstad, Oslo N-0601, Norway; 3 Department of Animal and Aquacultural Sciences, The Norwegian University of Life Sciences, Box 5003, 1432, Ås, Norway

## Abstract

**Background:**

Previous data suggested that intake of sodas and other acid beverages might be associated with increased levels of serum triglycerides, lowered HDL cholesterol, and increased formation of mono unsaturated fatty acids, which are the preferred ones for triglyceride synthesis. The present work is an extension of these studies.

**Methods:**

Thirty male rats were divided into 3 groups. All groups were given the same food, but various beverages: water (W), ammonium chloride, 200 mmol/L (AC), or sodium bicarbonate, 200 mmol/L (SB). Serum triglycerides, HDL cholesterol, and the fatty acid distribution in total serum lipids were determined. Delta9-desaturase in serum lipids was estimated by the ratio of palmitoleic to palmitic acid, and by the oleic/stearic acid ratio. Correlation and ANOVA were used to study associations and group differences.

**Results:**

After 3 weeks, the AC group had higher triglyceride concentration and higher Delta9 desaturase indexes, but lower serum HDL and body weight as compared with the SB and W groups. In each of the groups, the oleic acid/stearic acid ratio correlated positively with serum triglycerides; in the pooled group the correlation coefficient was r = 0.963, p<0.01.

**Conclusions:**

Rats ingesting ammonium chloride as compared with sodium bicarbonate responded with increased desaturase indexes, increased serum triglycerides, and lowered HDL cholesterol concentration, thereby possibly contributing to explain the increased triglyceride concentration previously observed in subjects with a frequent intake of acid beverages, such as sodas containing carbonic acid, citric acid, and phosphoric acid.

## Background

Sodas contain acids like carbonic acid, citric acid and phosphoric acid. In the cross sectional Oslo Health Study we reported that there was a positive association between intake of colas and serum triglycerides, irrespective of the presence or absence of sugar [1]. This observation raises the question of whether the acid content of colas might be causally implicated in the serum lipid effect. In support of this idea is our results obtained in a preliminary rat trial suggesting that animals ingesting various types of acid seemed to respond with increased fasting serum triglyceride concentration and decreased HDL cholesterol levels [2].

The serum triglyceride concentration in fasted animals is mainly carried in very low density lipoproteins (VLDL), which are synthesized and secreted in the liver [3]. VLDL- triglycerides, cholesterol esters and phospholipids preferably contain monounsaturated fatty acids, i.e. palmitoleic (C16:1 n7) and oleic (C18:1n6) acid [4]. The rate limiting enzyme for the synthesis of these fatty acids is stearoyl-CoA desaturase (SCD). Mice lacking SCD have reduced hepatic lipogenesis and lower plasma triglyceride concentration [5]. Accordingly, one mechanism by which an acid load might increase serum triglycerides could be stimulation of desaturase activities in the liver.

The present work is a follow-up and an extension of our previous preliminary study [2], achieved by increasing the group size, and using ammonium chloride to obtain an acid load. This agent has previously been used to produce metabolic acidosis [6]. We also included a group ingesting sodium bicarbonate, in an attempt to more easily reveal the acid effect upon the serum lipid concentration.

## Results

### Body weight and intake of food and beverages

There were no significant group differences in initial values of body weight, or in the zero time intakes of food and beverages (Table 1). After 3 weeks, the body weight was lower in the groups ingesting ammonium chloride or sodium bicarbonate as compared with water. Fluid intake after 3 weeks was higher in rats given sodium bicarbonate than in the other groups, which had similar fluid intakes.

**Table 1:** Body weight and intake of food and fluids in 3 groups of rats

	**Body weight (g)**		**Food intake (g)**		**Fluid intake (ml)**	
	**Initial**	**After 3 wk**	**Initial**	**After 3 wk**	**Initial**	**After 3 wk**
Water	143 ± 4^a^	254 ± 4	18.8 ± 0.6	21.2 ± 0.4	21.4 ± 1.5	22.8 ± 2.4
NH_4_Cl	141 ± 3	218 ± 4^b^	17.8 ± 0.5	20.6 ± 0.6	25.1 ± 2.0	25.5 ± 1.3
NaHCO_3_	142 ± 3	235 ± 4^b.c^	18.4 ± 0.5	22.9 ± 0.9	21.6 ± 1.5	56.3 ± 8.2^d^

Thirty male rats were divided into 3 equal groups and fed regular pellets for 3 weeks. One group was ingesting tap water, another was given a solution of ammonium chloride (200 mmol/L in the drinking water), and the third group ingested a solution of sodium bicarbonate (200 mmol/L in the drinking water). ^*a*^Mean ± SEM, n = 10. ^b^p <0.01 vs. water; ^c^p < 0.05 vs. NH_4_Cl; ^d^p < 0.001 vs. the other groups (ANOVA, with Tukey’s correction for multiple comparisons). Note that the food and fluid intake, for practical reasons, were recorded for 18 hours.

### Serum triglyceride and HDL cholesterol concentration in rats ingesting water, ammonium chloride, or sodium bicarbonate

**Figure 1:**
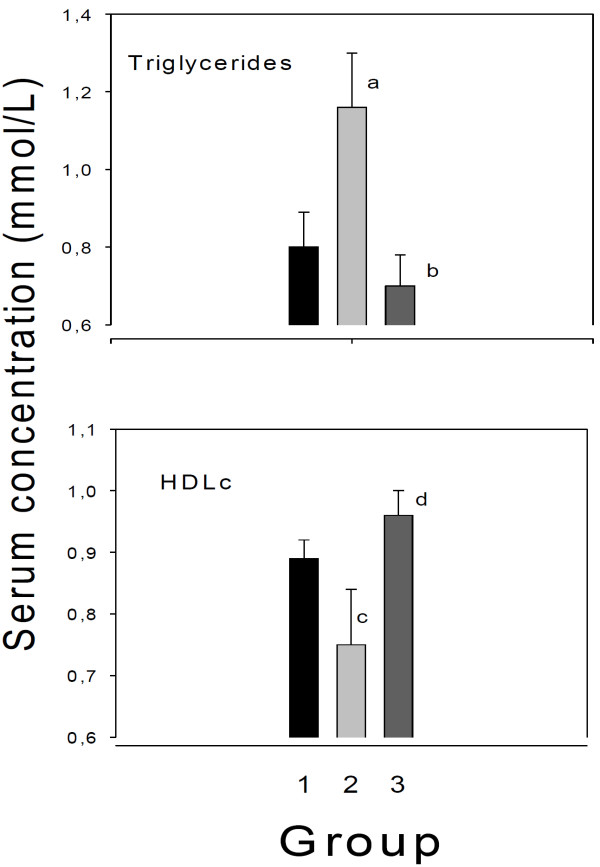
**Serum triglyceride and HDL cholesterol (HDLc) concentration in rats ingesting water, or solutions of ammonium chloride, or sodium bicarbonate.** Thirty male rats were divided into 3 equal groups and fed regular pellets for 3 weeks. One group was ingesting tap water (Group 1), another was given a solution of ammonium chloride (200 mmol/L in the drinking water, Group 2), and the third group ingested a solution of sodium bicarbonate (200 mmol/L in the drinking water, Group 3). ^a^p = 0.059 vs. group 1 (ANOVA, with Tukey correction for multiple comparisons); ^b^p = 0.013 vs. group 2; ^c^p = 0.023 vs group 1; ^d^p < 0.001 vs. group 2. Mean values ± SEM. Note broken axes.

For serum triglycerides (Figure 1, top panel), there was a significant between group effect (F = 5.2, p < 0.012). Using Tukey’s correction for multiple comparisons, there was a borderline significance (p = 0.059) for group 2 (ammonium chloride) vs. group 1 (water), and a significant difference (p = 0.013) between group 2 and group 3 (sodium bicarbonate), but no difference between group 1 and 3. Rats ingesting ammonium chloride had a significantly lower serum HDL cholesterol concentration than rats ingesting bicarbonate (p < 0.001, Figure 1, lower panel) or water (p = 0.023), but there was no significant differences in HDL between group 1 and 3. Thus, the group receiving ammonium chloride had significantly lower serum HDL concentration, and a borderline significantly higher serum triglyceride concentration than the control group, whereas there were significant differences in both variables when comparing the groups ingesting ammonium chloride or sodium bicarbonate.

### Estimates of Delta9 desaturase

Since the Delta9 desaturase activity is essential for catalyzing formation of the preferred fatty acids in serum triglycerides [4] we estimated the enzyme activity, using product/precursor ratios. As shown in Table 2, both of the Delta9 desaturase estimates were higher in rats ingesting ammonium chloride than in those ingesting water or a solution of sodium bicarbonate, but there was no difference in the enzyme estimates between the sodium bicarbonate and the water group.

**Table 2:** Estimates of Delta9 desaturase activity in rats ingesting various beverages

	**(16:1)/(16:0) ratio**	**(18:1)/(18:0) ratio**
Water	0.12 ± 0.01	0.97 ± 0.07
NH_4_Cl	0.17 ± 0.01^a^	1.36 ± 0.12^b^
NaHCO_3_	0.13 ± 0.01^c^	0.91 ± 0.07^d^

Thirty male rats were divided into 3 equal groups and fed regular pellets for 3 weeks. One group was ingesting tap water, another was given a solution of ammonium chloride (200 mmol/L in the drinking water), and the third group ingested a solution of sodium bicarbonate (200 mmol/L in the drinking water). Delta9 desaturase activity was estimated by the ratio of palmitoleic acid to palmitic acid in total lipid extract of serum, and by the oleic acid to stearic acid ratio. ^a^p = <0.001 vs. water (one-way ANOVA, with Tukey correction for multiple comparisons); ^b^p = 0.014 vs. water; ^c^p < 0.001 vs ammonium chloride; ^d^p = 0.005 vs. ammonium chloride. Mean values ± SEM.

### Relationship between the Delta9 desaturase estimate (18:1)/(18:0) ratio and serum triglycerides

**Figure 2:**
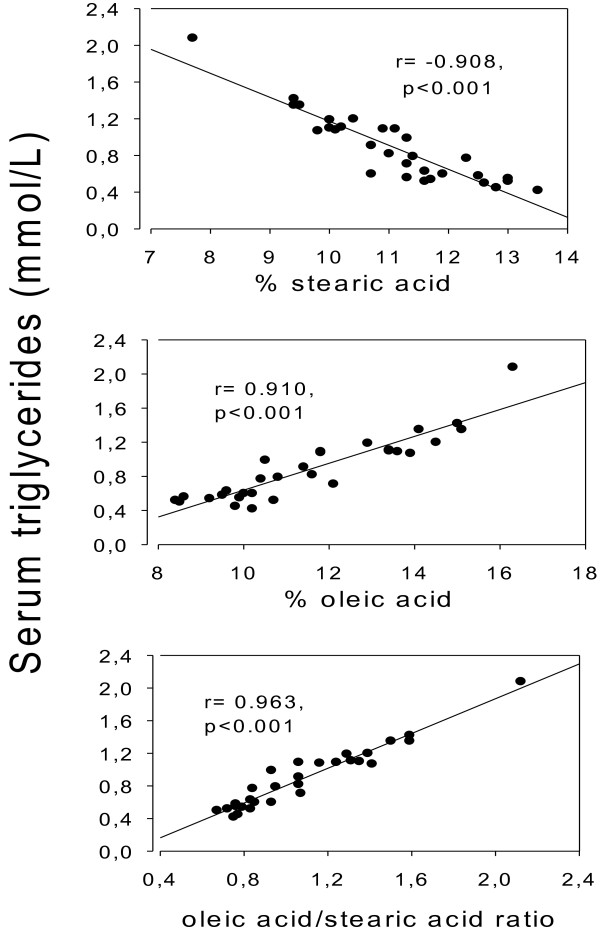
**Relationship between the Delta9 desaturase estimate (18:1)/(18:0) ratio and serum triglycerides.** Thirty male rats were divided into 3 equal groups and fed regular pellets for 3 weeks. One group was ingesting tap water (Group 1), another was given a solution of ammonium chloride (200 mmol/L in the drinking water, Group 2), and the third group ingested a solution of sodium bicarbonate (200 mmol/L in the drinking water, Group 3). The figure shows the values of the pooled sample (n = 30). Associations between serum triglycerides and stearic acid (top panel), oleic acid (middle panel), and the oleic/stearic acid ratio (lower panel) were all highly significant (p < 0.001).

In the pooled group, there was a negative association (r = −0.908, p < 001) between the triglyceride concentration in serum and percentage stearic acid in serum total lipids (Figure 2, top panel), but a positive association with per cent oleic acid (r = 0.910, p < 0.001; Figure 2, middle panel), and with the oleic acid/stearic acid ratio ( r = 0.963, p < 0.001; Figure 2, lower panel). These associations were also significant when studied in each of the 3 groups separately (not illustrated). For triglycerides vs. the oleic acid/stearic acid ratio: r = 0.939, p < 0.001; r = 0.980, p < 0.001; r = 0.893, p < 0.001 in the 3 groups, respectively. For triglycerides vs. stearic acid: r = −0.663, p = 0.036; r = −0.954, p < 0.001; r = −0.925, p < 0.001, and for triglycerides vs. oleic acid: r = 0.956, p < 0.001; r = 0.916, p < 0.001; r = 0.820, p = 0.004.

### Estimates of liver and kidney function

There were no significant group differences in liver transaminases (ASAT, ALAT) or in serum creatinine (results not shown).

## Discussion

The present results confirm our previous preliminary findings that an acid load seemed to increase the serum triglyceride concentration, and decrease serum HDL cholesterol, and that these changes were associated with increased value of indexes estimating the activity of Delta9 desaturase [2]. Thus, also the present approach to increase the acid load was followed by an altered serum lipid concentration.

Since an acid load seemed to increase the serum triglyceride concentration and lower HDL cholesterol, we would expect the opposite to occur after base ingestion. Although the mean values of serum lipids were according to this assumption, the changes did not reach statistical significance. This lack of effect might possibly be attributed to respiratory compensation of the alkalizating influence of bicarbonate.

It is well known that sucrose may increase the serum triglyceride concentration, and lower the serum high density lipoprotein cholesterol (HDL) concentration [7,8]. Additionally, soft drink intake has been shown to correlate positively with serum triglycerides, and negatively with HDL [9-12].

Soft drinks may contain organic acids, e.g. citric acid and carbonic acid. In addition, colas contain phosphoric acid [13,14]. The present results raise the question of whether intake of sucrose sweetened sodas also partly may stimulate triglycerides synthesis via acid dependent stimulation of Delta9 desaturase, caused both by the presence of various acids in sodas, and by the fructose moiety of sucrose giving increased formation and excretion of uric acid [15]. It has been reported previously that sucrose feeding of rats can increase the activity of desaturases [16].

Based upon results of a human population study we previously reported [1] that intake of colas was associated with increased serum triglyceride levels and reduced concentration of HDL, irrespective of whether the colas were with or without sugar. We subsequently reported a positive association between an index estimating the dietary acid load and indexes estimating risk of the metabolic syndrome [11,12], which may be a precursor of diabetes and cardiovascular diseases [17]. Based upon the present results we raise the question whether soft drink intake may influence serum lipids also via causing an acid load, which may stimulate fatty acid desaturation and formation of monounsaturated fatty acids, which are the preferred ones for hepatic triglyceride synthesis, the main VLDL component [3,4]. However, further studies are required to clarify whether this hypothesis is valid.

Additionally, the observed stimulatory influence of dietary sucrose upon lipogenesis might in general at least partly be attributed to an increased acid load, since the fructose moiety of sucrose may increase formation uric acid [15]. Thus, sucrose sweetened soft drinks may not only be a major carbohydrate source, but also contribute to the dietary acid load.

As referred to above, the concentration of serum triglycerides and HDL varies inversely after carbohydrate feeding. The present results would be in accordance with this finding. However, our results suggest that at least part of the sucrose influence upon serum lipids may be attributed to the acid forming potential of sucrose, since an agent providing acid seemed to cause this inverse relationship. Our data do not, however, clarify whether acid load reduces the HDL concentration secondary to raising serum triglycerides (VLDL), or via a more direct mechanism.

In rodents, intake of carbohydrates after fasting is accompanied by increased activity of hepatic Delta9-desaturase, as well as induction of mRNA for the enzyme [18]. Dephosphorylation of the transcription factor Carbohydrate Response Element-Binding Protein (ChREBP), and its translocation to the nucleus, has been shown to be involved in the carbohydrate activation of the desaturase [19]. Our results could provide an additional explanation for the increased Delta9 desaturase activity after sucrose intake, since the fructose moiety of sucrose is known to increase the acid load, as shown by increased urinary excretion of uric acid, calcium and oxalate [15]. Indeed, high intakes of acid soft drinks with sugar should have a particularly strong stimulating effect on hepatic lipogenesis, due to ample substrate supply, increased insulin release caused by the glucose moiety, and possibly by an acid dependent activation of desaturases. The fructose moiety of sucrose is rapidly converted to acetyl-CoA, the precursor of e.g. palmitic acid and stearic acid, which subsequently may be desaturated to palmitoleic and oleic acid by the acid stimulated Delta9-desaturase.

Intake of organic acids like citric acid will be metabolized to carbon dioxide and water, and the acid load of these acids may be regulated through the respiration. Thus, contrary to what might be expected, even fruits with a sour taste give a reduced acid load due to their high content of salts of organic acids. In contrast, an acid load caused by high intake of inorganic acids such as phosphoric acid will have to be regulated through renal mechanisms, implying increased excretion of H^+^, H_2_PO_4_^-^, and NH_4_^+^ ions.

Conceivably, various acids may act differently. However, in our previous report [2] we observed that widely varying acid beverages seemed to increase serum triglycerides and lower HDL cholesterol. In spite of this finding, the effect of the acid load on serum lipids, as studied in the present work, should not specifically relate to soft drink intake, since the typical acids found in soft drinks were not included in the present study. Therefore, the results may not necessarily be interpreted in support of our previous epidemiological findings of an association between soft drink intake and serum lipids.

Nevertheless, we have made a rough estimate of the acid load from intake of phosphoric acid in humans with a high intake of colas, and compared the estimate with the acid load from ammonium chloride intake in the present rat trial. We emphasize that, in our estimate, we only consider the approximate amount of dissociated acid ingested in drinking fluids. For the final biological influence, also other components in the diet, the buffer capacity of the body, and the intermittency of human cola drinking should be considered, as compared to the ad libitum drinking of the ammonium chloride solution in the rats. We do not know how the drinking pattern might possibly influence the final biological response of the acid load, for example whether high, but infrequent acid spikes might act differently from a lower, but more sustained acid load.

For phosphoric acid, assuming a concentration of about 0.5 g/L cola, and *K*_a1_ = 7.25 × 10^−3^, it may be estimated that the H^+^ concentration in colas, due to phosphoric acid, is approximately 3.5 mmol/L (pH = 2.5), representing about 50 μmol per kg body weight for a 70 kg person drinking 1 L of cola, or 5 μmoles for one glass of 100 mL.

NH_4_Cl is an acidic salt of ammonia (NH_3_) and HCl. When dissolved in water, a small portion of the produced NH_4_^+^ will further dissociate to hydrogen ions and ammonia. Using the dissociation constant, i.e. 5.69*10^-10^, the solution of 0.2 mol ammonium chloride/L will have a H^+^ concentration of 1.07*10^-5^ mol/L (pH = 5.0). Intake of 25 ml of this solution per day would accordingly give the rat an acid load of about 1.34 μmol H^+^ per kg body weight per day. This value is low compared to the one related to phosphoric acid intake in human subjects ingesting large amounts of colas. In this context it should be remembered that in humans, phosphoric acid adds to other sources of acid load, related to both diet components (e.g. high protein intake) and air pollution. Additionally, the rat experiment lasted for 3 weeks only, whereas the acid load in humans may continue for several decades.

### Significance of desaturase activation by increasing the acid load

Human epidemiological studies have shown that fatty acid desaturase indexes such as the ratio of C16:1n7 to C16:0, an estimate of stearoyl-CoA desaturase, may predict cardiovascular mortality [20] and inhibition of the enzyme may be associated with increased insulin sensitivity [21]. Studies in mice lacking stearoyl-CoA desaturase (SCD) seem to offer an explanation of the epidemiological findings [5,22-25]. Mice lacking the enzyme do not have the ability to form monounsaturated fatty acids which are significant constituents of tissue lipids and serum lipoproteins. These animals have reduced hepatic lipogenesis, lowered plasma triglyceride levels, and increased insulin sensitivity compared with their normal counterparts. These studies indicate that desaturases are important in metabolic control. Anticipating that desaturase inhibition is important also in man, our results could imply that an increased dietary acid load, possibly obtained by for example high intake of sugar-sodas, might promote major lifestyle diseases, such as obesity, diabetes and cardiovascular diseases via stimulation of liver desaturases. We emphasize that studies in man are required to substantiate this hypothesis. In this context it should also be kept in mind a study in hyperlipidemic mice suggesting that inhibition of stearoyl-CoA desaturase 1 may promote aortic atherosclerosis in spite of protecting against diet-induced obesity and insulin resistance [26].

The highly significant positive association between percentage oleic acid and serum triglyceride concentration is in accordance with the view that the oleic acid is the preferred substrate for triglyceride formation [4]. This contention is further substantiated by the positive association between the Delta9 desaturase estimate and the serum triglyceride concentration (Figure 2).

We previously suggested for the first time that an increased acid load may act as a physiological enhancer of the activity of fatty acid desaturases [2]. The present results would seem in keeping with this suggestion. However, different acids may have different effects on the delta9 desaturase, but we have no data to substantiate this possibility.

Further studies are required to explore whether the presented hypothesis of an association between the acid load, desaturase activity and serum lipids is valid. Among such studies could be studies related to progression of atherosclerosis as influenced by increased acid load, free radical generation, lipid peroxidation, and cytokine profile, and also whether the profile of unsaturated fatty acids in general might be modified, in plasma, erythrocytes, blood vessels and various tissues.

## Conclusions

We suggest that an increased acid load may contribute to increased desaturase activity, followed by increased formation of monounsaturated fatty acids which govern the synthesis of hepatic triglycerides and probably other compound lipids. These events would in turn result in increased hepatic synthesis and output of VLDL, to be measured for example as an increased fasting serum triglyceride concentration. Our data raise the question of whether an increase dietary acid load, possibly caused by frequent intake of sucrose and/or acid soft drinks, may promote obesity, diabetes and cardiovascular diseases also by creating a low-grade metabolic acidosis which in turn would increase the activity of fatty acid desaturases and subsequent increased lipid formation. However, further studies in man are needed to confirm this hypothesis.

## Methods

### Ethical approval

The present trial in rats was performed with the approval of the Regional Norwegian Ethics Committee, and the experimental research followed internationally recognized guidelines.

### Feeding

Thirty male rats (Mol:Wist, L1, Skensved, Denmark) were kept in-house for a one week acclimatisation before the diet trial. Throughout the study period the rats were fed ad libitum maintenance rat pellets RM1 from Special Diets Services, England (2.7% crude fat, 14.4% crude protein, 4.7% crude fibre and 6.0% crude ash). Distribution of fatty acids in the pellet diet (g/100 g pellets) was: lauric acid 0.02; myristic acid 0.14; palmitic acid 0.31; stearic acid 0.04; myristoleic acid 0.2; palmitoleic acid 0.09; oleic acid 0.77; linoleic acid 0.69; linolenic acid 0.06; arachidonic acid 0.13. The animals were divided into three groups with ten rats in each group and given different beverages (all given ad libitum): Group 1) tap water (control group); Group 2) ammonium chloride (200 mmol/L; 1% ) in the drinking water; Group 3) sodium bicarbonate (200 mmol/L; 1.7% ) in the drinking water. Data for body weight, intake of food and consumed volumes of the various beverages, and volume of excreted urine were collected after three weeks when ending the trial. For practical reasons, measurements of the intake of food and fluids were carried out during 18 hours.

### Blood and tissue sampling

After a 4–6 hour fast, venous blood was collected from the right dorso-lateral tail vein after six weeks (end of study), using heparin-moistened syringes. Blood samples were centrifuged at 1750 x g for 10 min. and the supernatant was collected and frozen at −70°C. The animals were killed by an overdose of pentobarbital (10 mg/ml) intraperitoneally.

### Determination of serum total cholesterol, HDL cholesterol and triglycerides

Serum total cholesterol was determined with Cholesterol CHOP-PAP kit (12016630 122, Roche/Hitachi), and HDL-cholesterol using an enzymatic HDL-Cholesterol kit (Biomed Labordiagnostik GmbH, Germany). Triglycerides in serum was determined using Triglycerides GPO-PAP kit (12016648 122, Roche/Hitachi).

### Determination of fatty acids

The fatty acid profile was determined in serum total lipids. The lipids were extracted using n-butanol Diheptadecanoyl-glycerophospho ethanolamine and butylated hydroxytoluene (Sigma Chemical, United Kingdom) were added as internal standard and antioxidant, respectively. The lipids were transmethylated and fatty acid methyl esters quantified as mg fatty acid/g tissue, using gas liquid chromatography on a SP2330 column (Supelco Inc., Bellefonte, PA). A normal human serum sample was included to assess analytical performance. The results of the measurements are presented as weight percentage of total fatty acids. The following 14 fatty acids in serum and liver total lipids were analyzed: Myristic acid (14:0), palmitic acid (16:0), palmitoleic acid (16:1 n7), stearic acid (18:0), oleic acid (18:1 n9), linoleic acid (18:2 n6), linolenic acid (18:3 n3), eicosenoic acid (20:1 n9), eicosadienoic acid (20:2 n6), arachidonic acid (20:4 n6), eicosatrienoic (20:3 n3), EPA (20:5 n3), erucic acid (22:1 n9), DHA (22:6 n3). The day to day coefficient of variation (n = 28) for 18:0, 18:1 n9, 18:2 n6, 20:4 n6, 20:5 n3 and 22:6 n3 was 5.2, 6.2, 6.6, 9.6, 10.0, and 11.6% , respectively. The main focus in the present work was on palmitic-, palmitoleic-, stearic-, and oleic acid, serving as components of Delta9 desaturase indexes.

### Estimates of fatty acid desaturases

To estimate Delta9-desaturase, we used the (16:1)/ (16:0) and (18:1)/ (18:0) ratios in total serum lipids. We previously reported that in fasted rats, corresponding indexes based upon fatty acids in total serum lipids and in the phospholipid fraction correlated positively [2]. Since we observed that the desaturase indexes calculated from fatty acids in total lipids in serum and liver correlated positively, in the present work we estimated Delta9 desaturase using fatty acids in serum total lipids.

### Statistical analysis

The influence of the various beverages upon serum lipids and desaturase indexes was assessed by correlation analyses (Pearson), by one-way ANOVA with Tukey’s correction for multiple comparisons. We used SPSS 19.0 for the analyses, and Sigma Plot 2001 to produce the figures. A significance level of 0.05 was accepted. The results are given as mean values ± SEM.

## Competing interests

The authors declare that they have no competing interests.

## Authors’ contributions

ATH conceived and designed the study, analyzed and interpreted the data, and wrote the article. MSHL contributed substantially to the interpretation of data and revising it critically for important intellectual content. AH contributed substantially to the interpretation of data and revising it critically for important intellectual content. All the authors approved the final version to be published.

## References

[B1] Høstmark AT Tomten SE Cola intake and serum lipids in the Oslo health study Appl Physiol Nutr Metab 2009 34 901 906 10.1139/H09-094 19935852

[B2] Høstmark AT Lunde MSH Eilertsen E Does an acid load promote liver desaturases and increase serum lipids? Clin Rev Opinions 2010 2 8 16

[B3] Mayes PA Murray RK, Granner DK, Mayes PA, Rodwell VW Lipid transport and storage Harper’s Biochemistry 2000 25 New York: McGraw-Hill 268 284

[B4] Nakamura MT Nara TY Structure, function, and dietary regulation of delta6, delta5, and delta9 desaturases Ann Rev Nutr 2004 24 345 376 10.1146/annurev.nutr.24.121803.063211 15189125

[B5] Dobrzyn A Ntambi JM Stearoyl-CoA desaturase as a new drug target for obesity treatment Obes Rev 2005 6 169 174 10.1111/j.1467-789X.2005.00177.x 15836467

[B6] Chang-Sun K Dong-Ho P Effects of chronic NH4Cl dosage and swimming exercise on bone metabolic turnover in rats J Physiol Anthropol Appl Human Sci 2005 24 6 595 600 10.2114/jpa.24.595 16377944

[B7] Høstmark AT Blom PC Previous exercise nullifies the plasma triacylglycerol response to repeated fructose ingestion in young men Acta Physiol Scand 1985 125 553 554 10.1111/j.1748-1716.1985.tb07755.x 4083051

[B8] Archer SL Liu K Dyer AR Ruth KJ Jacobs DR Jr Van HL Hilner JE Savage PJ Relationship between changes in dietary sucrose and high density lipoprotein cholesterol: the CARDIA study. Coronary artery risk development in young adults Ann Epidemiol 1998 8 433 438 10.1016/S1047-2797(98)00007-6 9738689

[B9] Dhingra R Sullivan L Jacques PF Wang TJ Fox CS Meigs JB D’Agostino RB Gaziano JM Vasan RS Soft drink consumption and risk of developing cardiometabolic risk factors and the metabolic syndrome in middle-aged adults in the community Circulation 2007 116 480 488 10.1161/CIRCULATIONAHA.107.689935 17646581

[B10] Merchant AT Anand SS Kelemen LE Vuksan V Jacobs R Davis B Teo K Yusuf S Carbohydrate intake and HDL in a multiethnic population Am J Clin Nutr 2007 85 225 230 17209200 10.1093/ajcn/85.1.225

[B11] Høstmark AT The Oslo health study: soft drink intake is associated with the metabolic syndrome Appl Physiol Nutr Metab 2010 35 635 642 10.1139/H10-059 20962919

[B12] Høstmark AT The Oslo health study: a dietary index estimating high intake of soft drinks and low intake of fruits and vegetables was positively associated with components of the metabolic syndrome Appl Physiol Nutr Metab 2010 35 816 825 10.1139/H10-080 21164553

[B13] Jensdottir T Holbrook P Nauntofte B Buchwald C Bardow A Immediate erosive potential of cola drinks and orange juices J Dent Res 2006 85 226 230 10.1177/154405910608500304 16498068

[B14] Kapicloglu S Baki AH Tekelioglu Y Araz K The effect of cola consumption on oral mucosa in rats Dis Esophagus 2000 2000 13 69 71 11005335 10.1046/j.1442-2050.2000.00082.x

[B15] Taylor EN Curhan GC Fructose consumption and the risk of kidney stones Kidney Int 2008 73 207 212 10.1038/sj.ki.5002588 17928824

[B16] Brenner RR Rimoldi OJ Lombardo YB Gonzalez MS Bernasconi AM Chicco A Basabe JC Desaturase activities in rat model of insulin resistance induced by a sucrose-rich diet Lipids 2003 38 733 742 10.1007/s11745-003-1121-x 14506836

[B17] Wilson PWF D’Agostino RB Parise H Sullivan L Meigs JB Metabolic syndrome as a precursor of cardiovascular disease and type 2 diabetes mellitus Circulation 2005 112 3066 3072 10.1161/CIRCULATIONAHA.105.539528 16275870

[B18] Ntambi JM The regulation of stearoyl-CoA desaturase (SCD) Progr Lipid Res 1995 34 139 150 10.1016/0163-7827(94)00010-J 7480063

[B19] Latasa MJ Moon YS Kim KH Sul HS Nutritional regulation of the fatty acid synthase promoter in vivo: sterol regulatory element binding protein functions through an upstream region containing a sterol regulatory element Proc Natl Acad Sci 2000 97 10619 10624 10.1073/pnas.180306597 10962028 PMC27074

[B20] Warensjo E Sundstrom J Vessby B Cederholm T Riserus U Markers of dietary fat quality and fatty acid desaturation as predictors of total and cardiovascular mortality: a population-based prospective study Am J Clin Nutr 2008 88 203 209 18614742 10.1093/ajcn/88.1.203

[B21] Corpeleijn E Feskens EJM Jansen EHJM Mensink M Saris WHM De Bruin TWA Blaak EE Improvements in glucose tolerance and insulin sensitivity after lifestyle intervention are related to changes in serum fatty acid profile and desaturase activities: the SLIM study Diabetologia 2006 49 2392 2401 10.1007/s00125-006-0383-4 16896932

[B22] Dobrzyn A Ntambi JM The role of stearoyl-CoA desaturase in body weight regulation Trends Cardiovasc Med 2004 14 77 81 10.1016/j.tcm.2003.12.005 15030794

[B23] Dobrzyn A Ntambi JM The role of stearoyl-CoA desaturase in the control of metabolism Prostagl Leukotr Essent Fatty Acids 2005 73 35 41 10.1016/j.plefa.2005.04.011 15941655

[B24] Dobrzyn A Dobrzyn P Lee SH Miyazaki M Cohen P Asilmaz E Hardie DG Friedman JM Ntambi JM Stearoyl-CoA desaturase-1 deficiency reduces ceramide synthesis by downregulating serine palmitoyltransferase and increasing beta-oxidation in skeletal muscle Am J Physiol Endocrinol Metab 2005 288 E599 E607 15562249 10.1152/ajpendo.00439.2004

[B25] Dobrzyn P Ntambi JM Dobrzyn A Stearoyl-CoA desaturase: A novel control point of lipid metabolism and insulin sensitivity Eur J Lipid Sci Techn 2008 110 93 100 10.1002/ejlt.200700249

[B26] Brown JM Chung S Sawyer JK Degirolamo C Alger HM Nguyen T Zhu X Duong MN Wibley AL R S Matthew AD Kelley K Wilson MD Kent C Parks JS Rudel LL Inhibition of stearoyl-coenzyme a desaturase 1 dissociates insulin resistance and obesity from atherosclerosis Circulation 2008 118 1467 1475 10.1161/CIRCULATIONAHA.108.793182 18794388 PMC2716169

